# Supplementary material for: Biological process activity transformation of single cell gene expression for cross-species alignment
Source: Nat Commun. 2019 Oct 25;10:4899. doi: 10.1038/s41467-019-12924-w (PMC6814736; doi:10.1038/s41467-019-12924-w)
Supplement: Supplementary file 2 — Description of Additional Supplementary Files [file 41467_2019_12924_MOESM2_ESM.pdf]

Description of Additional Supplementary Files

File Name: Supplementary Data 1
Description: Sorted combined GO biological process (C5) and immunologic (C7) gene set names according to differential activity in B-cells, T-cells and monocytes in Chromium 10x dataset.

File Name: Supplementary Data 2
Description: Sorted combined GO biological process (C5) and immunologic (C7) gene set names according to differential activity in B-cells and T-cells in [15] dataset.

File Name: Supplementary Data 3
Description: Sorted combined GO biological process (C5) and immunologic (C7) gene set names according to differential activity in monocytes, pDCs and cDCs in [5] dataset.
