# Supplementary material for: Biological process activity transformation of single cell gene expression for cross-species alignment
Source: Nat Commun. 2019 Oct 25;10:4899. doi: 10.1038/s41467-019-12924-w (PMC6814736; doi:10.1038/s41467-019-12924-w)
Supplement: Supplementary file 6 — Reporting Summary [file 41467_2019_12924_MOESM6_ESM.pdf]

nature research

Corresponding author(s): HONGXU DING
JOSHUA M. STUART

Last updated by author(s): Sep 27, 2019

Reporting Summary

Nature Research wishes to improve the reproducibility of the work that we publish. This form provides structure for consistency and transparency in reporting. For further information on Nature Research policies, see Authors & Referees and the Editorial Policy Checklist.

Statistics

For all statistical analyses, confirm that the following items are present in the figure legend, table legend, main text, or Methods section.

n/a	Confirmed	
☐	☒	The exact sample size (*n*) for each experimental group/condition, given as a discrete number and unit of measurement
☐	☒	A statement on whether measurements were taken from distinct samples or whether the same sample was measured repeatedly
☐	☒	The statistical test(s) used AND whether they are one- or two-sided<br>*Only common tests should be described solely by name; describe more complex techniques in the Methods section.*
☐	☒	A description of all covariates tested
☐	☒	A description of any assumptions or corrections, such as tests of normality and adjustment for multiple comparisons
☐	☒	A full description of the statistical parameters including central tendency (e.g. means) or other basic estimates (e.g. regression coefficient) AND variation (e.g. standard deviation) or associated estimates of uncertainty (e.g. confidence intervals)
☐	☒	For null hypothesis testing, the test statistic (e.g. *F*, *t*, *r*) with confidence intervals, effect sizes, degrees of freedom and *P* value noted<br>*Give P values as exact values whenever suitable.*
☒	☐	For Bayesian analysis, information on the choice of priors and Markov chain Monte Carlo settings
☒	☐	For hierarchical and complex designs, identification of the appropriate level for tests and full reporting of outcomes
☒	☐	Estimates of effect sizes (e.g. Cohen's *d*, Pearson's *r*), indicating how they were calculated

Our web collection on statistics for biologists contains articles on many of the points above.

Software and code

Policy information about availability of computer code

Data collection	CRAN R package msigdbr (6.2.1) was used for collecting gene sets.
Data analysis	Bioconductor R package viper (1.14.0) was used for BPA inference.

For manuscripts utilizing custom algorithms or software that are central to the research but not yet described in published literature, software must be made available to editors/reviewers. We strongly encourage code deposition in a community repository (e.g. GitHub). See the Nature Research guidelines for submitting code & software for further information.

Data

Policy information about availability of data

All manuscripts must include a data availability statement. This statement should provide the following information, where applicable:

- Accession codes, unique identifiers, or web links for publicly available datasets
- A list of figures that have associated raw data
- A description of any restrictions on data availability

GTEx bulk RNA sequencing profiles can be found from the website: https://gtexportal.org/home/. We downloaded the provided normalized expression profiles and log-transformed them into log2(RPKM+1) for downstream analysis.

Mouse and human esophageal epithelium normalized bulk RNA-Seq expression profiles: https://www.ncbi.nlm.nih.gov/geo/query/acc.cgi?acc=GSE116272.

scRNA-Seq profiles for the human PBMC dataset were taken from healthy donors generated using 10x Genomics V2 and V1 chemistry and available from: https://support.10xgenomics.com/single-cell-gene-expression/datasets/2.0.1/pbmc4k, https://support.10xgenomics.com/single-cell-gene-expression/datasets/1.1.0/pbmc3k. We downloaded the provided UMI counts and normalized by the sequencing depth as log2(TPM+1) for downstream analysis.

scRNA-Seq profiles for the human preimplantation embryo dataset, including time point: https://www.ebi.ac.uk/arrayexpress/experiments/E-MTAB-3929/. We

downloaded the provided normalized expression profiles and log-transformed them into log2(RPKM+1) for downstream analysis.

scRNA-Seq profiles for the mouse embryonic stem cell dataset: https://www.ncbi.nlm.nih.gov/geo/query/acc.cgi?acc=GSE65525. We downloaded the provided normalized expression profiles and log-transformed them into log2(TPM+1) for downstream analysis.

scRNA-Seq profiles for the zebrafish embryo dataset: https://www.ncbi.nlm.nih.gov/geo/query/acc.cgi?acc=GSE66688. We downloaded the provided normalized expression profiles and log-transformed them into log2(TPM+1) for downstream analysis.

scRNA-Seq profiles for the human and mouse early embryos, including time point annotations: https://www.nature.com/articles/nature12364. We downloaded the provided normalized expression profiles and log-transformed them into log2(RPKM+1) for downstream analysis.

scRNA-Seq profiles for the human monocytes and dendritic cells, including cell type annotation: http://science.sciencemag.org/content/356/6335/eaah4573. We downloaded the provided normalized expression profiles and log-transformed them into log2(TPM+1) for downstream analysis.

Tabula Muris datasets: https://www.nature.com/articles/s41586-018-0590-4. We downloaded the provided counts of spleen and thymus datasets and normalized by the sequencing depth as log2(CPM+1) for downstream analysis.

Field-specific reporting

Please select the one below that is the best fit for your research. If you are not sure, read the appropriate sections before making your selection.

☒ Life sciences ☐ Behavioural & social sciences ☐ Ecological, evolutionary & environmental sciences

For a reference copy of the document with all sections, see nature.com/documents/nr-reporting-summary-flat.pdf

Life sciences study design

All studies must disclose on these points even when the disclosure is negative.

Sample size	NA
Data exclusions	NA
Replication	NA
Randomization	NA
Blinding	NA

Reporting for specific materials, systems and methods

We require information from authors about some types of materials, experimental systems and methods used in many studies. Here, indicate whether each material, system or method listed is relevant to your study. If you are not sure if a list item applies to your research, read the appropriate section before selecting a response.

Materials & experimental systems

n/a	Involved in the study	
☒	☐	Antibodies
☒	☐	Eukaryotic cell lines
☒	☐	Palaeontology
☒	☐	Animals and other organisms
☒	☐	Human research participants
☒	☐	Clinical data

Methods

n/a	Involved in the study	
☒	☐	ChIP-seq
☒	☐	Flow cytometry
☒	☐	MRI-based neuroimaging
